# Social Media Use, Professionalism, and Professional Identity Among Medical Students and Early-Career Health Care Graduates in Sudan: Cross-Sectional Survey Study

**DOI:** 10.2196/89260

**Published:** 2026-08-25

**Authors:** Fatima Elbasri Abuelgasim Mohammed, Enas Osama Hassan Omer, Ammar Elgadi, Ola Rashid Eltayeb Mamoun, Mohamed Shawgi Yassin Alsayed, Saad Elsayed, Ragheda Gamal Abdelrafi Musaad, Salma Sleak Alam, Mohamed Adam, Bayadir Adam Mohamed Adam, Sara Isam Eldin Ibrahim Elshafie, Mohanad Mustafa Mohamed Elamin, Omer Abdallah Mohamedelhassan Mohamed

**Affiliations:** 1 Faculty of Medicine, University of Khartoum, Alqast Street, Khartoum, 1111, Sudan, 249 121006598, 249 121006598; 2 Faculty of Medicine, University of Bahri, Khartoum, Sudan; 3 Faculty of Medicine, Al-neelain University, Khartoum, Sudan; 4 Faculty of Medicine, Elrazi University, Khartoum, Sudan; 5 Faculty of Pharmacy, Sudan University of Science and Technology, Khartoum, Sudan; 6 Faculty of Medicine, University of Medical Sciences and Technology, Khartoum, Sudan; 7 National Ribat University, Khartoum, Sudan; 8 Community Medicine Department, Merowe University of Technology, Merowe, Sudan

**Keywords:** social media, digital professionalism, professional identity formation, medical students, early-career doctors

## Abstract

**Background:**

Social media has become deeply embedded in medical training, yet its influence on professionalism and the developing professional identity of medical students and early-career doctors remains insufficiently understood, particularly in non-Western contexts.

**Objective:**

This study aimed to assess social media usage patterns, attitudes toward digital professionalism, related behaviors and experiences, and perceived benefits and risks among medical students and early-career health care graduates in Sudan, and to identify demographic and behavioral factors associated with key online behaviors and outcomes.

**Methods:**

A cross-sectional online survey was conducted from June 2025 to August 2025 among 432 medical students, interns, early graduates, and residents in Sudan, recruited through convenience sampling. A structured, self-administered questionnaire developed specifically for this study assessed social media usage patterns, attitudes toward digital professionalism, related behaviors and experiences, and perceived benefits and risks. Descriptive statistics, chi-square tests, and multivariable logistic regression were performed using R software (version 4.4.2; R Foundation for Statistical Computing).

**Results:**

Of the 432 participants, 226 (52.3%) were female participants, and 343 (79.4%) spent at least 3 hours daily on social media. Attitudes toward digital professionalism were generally positive: 225 (52.1%) participants agreed or strongly agreed that their online presence reflects their professional identity, and 390 (90.3%) considered maintaining a professional image is important. Nearly half of the participants (n=199, 46.1%) had modified or removed posts for professional reasons. The most reported benefits were access to updated medical knowledge (n=321, 74.3%) and career development (n=260, 60.2%), while common concerns involved misinformation (n=287, 66.4%), negative public perception (n=199, 46.1%), and confidentiality breaches (n=198, 45.8%). Female participants were more likely to report heavy daily use of 5 hours or more (109/226, 48.2% vs 70/206, 33.9%; *P*=.004). In multivariable analysis, male sex (odds ratio 2.12, 95% CI 1.36‐3.33; *P*<.001) and posting health care–related content (odds ratio 2.64, 95% CI 1.58‐4.52; *P*<.001) independently predicted experiencing a negative outcome on social media, whereas confidence in distinguishing appropriate content showed no significant demographic variation.

**Conclusions:**

Medical students and early-career doctors in Sudan engage heavily with social media and recognize both its value and its risks to professional identity. These findings highlight the need for structured digital professionalism training and culturally sensitive guidelines to support safe and responsible online engagement.

## Introduction

### Background

As digital environments become embedded in the culture of medicine, important questions remain regarding how these online interactions influence the development of a student’s professional identity—a hallmark of medical formation. Understanding this dynamic has become a global priority, as educators and regulators attempt to balance the opportunities of online engagement with the preservation of professionalism and public trust.

Professional identity formation (PIF) is the process by which medical students internalize the values, behaviors, and norms of the medical profession and come to “think, act, and feel like a physician” [1]. It is both an individual and a social process, shaped by educational structures, mentorship, institutional culture, and peer interactions. Both PIF frameworks and social identity theory propose that identity develops through an individual’s integration into a professional community [1]. Social media has added a new dimension to this process: it reshapes how members of the medical community interact within the framework of professionalism, giving rise to identities that are increasingly shaped by digital visibility and online participation [1,2]. If used effectively, social media platforms such as X (formerly Twitter), LinkedIn, Instagram, and YouTube can facilitate networking and knowledge and experience sharing [2]. In addition, medical professionals can use social media to advocate for patients’ rights and raise awareness regarding emerging health issues [2]. Furthermore, social media can be used to create meaningful learning experiences, facilitate collaboration among peers, and foster reflective discussions [3,4]. However, social media platforms can predispose medical students and early-career doctors to ethical dilemmas and reputational damage. Many debates about these platforms’ impact on confidentiality, misinformation, and the blurring of boundaries have raised questions about the extent to which individuals should conduct themselves professionally on these platforms [5,6].

Different studies have discussed the connection between social media and professional identity. For example, Garner and O’Sullivan [7] reported that medical students in the United Kingdom expressed concern over practices among their colleagues in using images on Facebook that might impact their professional identity. White et al [8] reported that the majority of students who used Facebook identified content about alcohol or drug use, obesity, criticism of others, and sexual content as unprofessional behaviors, although 44% of them had seen a colleague posting about these negative behaviors. It has become inevitable that medical students and early-career doctors become aware of the implications that social media has on ethics and professionalism [9]. However, a large majority of the existing literature is grounded in Western educational and cultural contexts, wherein norms of self-presentation and professional autonomy differ markedly from those in collectivist societies. In many regions, including the Middle East and North Africa, cultural expectations surrounding modesty, authority, and social reputation interact strongly with perceptions of professionalism. Thus, the negotiation of identity in digital spaces may be considerably more complex, as students must reconcile globalized norms of visibility and advocacy with local ethical frameworks and institutional hierarchies [10]. Taken together, prior studies establish that unprofessional online behavior is common and consequential, and that social media meaningfully shapes professional identity; however, this evidence base offers little evidence from African or Middle Eastern settings, and, to our knowledge, no study to date has examined how medical students and early-career doctors in Sudan perceive the relationship between social media and their professional identity.

Understanding these nuances is important, as PIF is context-dependent. What counts as self-expression or advocacy in one setting may be considered unprofessional or inappropriate in another. In the absence of empirical data that reflect these sociocultural differences, the creation of social media guidelines or educational interventions will fall victim to misalignment with local realities [10].

In addition, it is important to highlight 2 major concerns. First, while many medical institutions globally have issued guidelines on digital professionalism, few provide comprehensive training that helps students and doctors critically reflect on their digital identities [11]. Second, there is limited empirical understanding of how cultural context mediates students’ and doctors’ online experiences and how these, in turn, influence their developing sense of professionalism.

Digital professionalism needs to be taught not as a set of rules, but as a reflective competency—a practice of judgment, accountability, and self-awareness. Formal curricula, institutional policies, and frameworks for mentoring now need to be developed to help students manage their digital presence responsibly and authentically. Several studies have found that interactive workshops, reflective exercises, and faculty modeling can significantly enhance students’ awareness of professional behavior online [11-13].

### Objectives

The aim of this study was to investigate the perspectives of medical students and early-career health care graduates in Sudan regarding the influence of social media on medical professional identity. Specifically, the study sought to (1) describe social media usage patterns; (2) assess attitudes toward professionalism and professional identity online; (3) document professionalism-related behaviors, experiences, and perceived benefits and risks; and (4) identify demographic and behavioral factors associated with heavy social media use, health care–related content engagement, confidence in posting appropriateness, and negative online outcomes.

## Methods

### Study Design and Setting

A cross-sectional, institution-based study was conducted from June 2025 to August 2025 among medical students and early-career health professionals in Sudan.

### Study Population and Sampling

We targeted medical students from the first to the final year of study. In addition, we included early-career health professionals (interns, early graduates in practice, and residents) who had graduated within the previous 10 years. Because there is no single, universally accepted definition of the early-career phase, different bodies apply varying cutoffs, we operationalized “early career” as within 10 years of graduation, consistent with Rotenstein et al [14], who characterize the early-career phase as approximately the first decade in which physicians establish themselves in independent practice and develop their professional identity. Convenience sampling was used, and participants were reached through an anonymous online questionnaire (Google Forms). The survey link was open to anyone who received it and was distributed via medical social media groups and WhatsApp groups dedicated mainly to medical students and health care professionals. Participation was voluntary, and no incentives were offered.

### Sample Size Calculation

The sample size was calculated using the following formula: n = (z² × p × q)/d², where n is the sample size, z is the *z* score (1.96 for a 95% confidence level), p is the expected proportion (0.5), q is 1−p, and d is the desired margin of error (0.05), yielding a minimum required sample size of 384 participants.

### Data Collection Tool and Procedure

An anonymous, structured, self-administered questionnaire in English was developed specifically for this study to assess medical students’ and early medical graduates’ social media use, perceptions of digital professionalism, and experiences related to professional identity. The items were newly constructed by the research team rather than adapted from a previously validated instrument, and their content was informed by the existing literature on digital professionalism and online identity among medical students and health care professionals [2,7,8,12]. The instrument consisted of 6 sections. The first section captured participant demographics (6 items), including age, sex, academic level, and current status.

The *Perceptions of Professionalism and Professional Identity on Social Media* section consisted of 5 attitudinal items. These items assessed participants’ views on the professional implications of their social media presence, including whether their online activity reflects their identity as a health care professional, the importance of maintaining a professional image, the perceived influence of social media on public trust, the potential enhancement of professional reputation through online engagement, and their confidence in distinguishing appropriate from inappropriate online content. Because these items represent conceptually related attitudes toward digital professionalism, they were evaluated psychometrically. Responses were coded on ordinal or binary scales according to established scoring rules. The construct validity of the scale was assessed using exploratory factor analysis (EFA; Multimedia Appendix 1), and Cronbach α was subsequently calculated for the initial 5-item scale and for the final 4-item scale retained after factor analysis, with item-total statistics used to evaluate the contribution of each item (Multimedia Appendix 2).

The *Experiences and Behaviors* domain consisted of 7 items that assessed participants’ real-world actions and encounters related to social media use within health care contexts. These items included following health care–related pages, posting health care–related content, modifying or removing posts for professional reasons, experiencing negative outcomes related to social media activity, maintaining separate personal and professional accounts, encountering inappropriate content shared by health care professionals, and using social media for various educational or professional purposes. Collectively, these items capture diverse behavioral patterns and experiential events, ranging from usage habits to corrective behaviors and exposure to unprofessional content. Because these items represent independent experiences rather than indicators of a single latent construct, they are not expected to correlate strongly. In addition, the items vary in format, some binary (yes or no), others categorical, and one functioning as a multiresponse checklist, making internal consistency measures such as Cronbach α methodologically inappropriate. Therefore, reliability analysis was not conducted for this domain.

Two additional checklist items were included to assess participants’ knowledge and awareness regarding social media use in health care. These questions asked respondents to identify the benefits they associate with using social media as health care students or professionals and the risks they perceive in a health care context. As checklist-style items reflecting knowledge rather than attitudes or behaviors, they were analyzed descriptively and were not included in reliability testing. The survey was reported in accordance with the CHERRIES (Checklist for Reporting Results of Internet E-Surveys) guidelines (Checklist 1).

The questionnaire was administered via Google Forms across five pages: (1) informed consent; (2) demographic characteristics, including selection of career level (undergraduate or postgraduate); (3) items for medical students (eg, university and academic year); (4) items for practicing doctors (eg, public or private hospital); and (5) the attitude, behavior, and checklist items described above. All items were mandatory, so incomplete questionnaires could not be submitted, and respondents were unable to change their answers after submission. Items and response options were presented in a fixed order for all respondents, without randomization, and no adaptive questioning was used. No technical measures (cookies, IP address checks, or log file analysis) were applied to prevent duplicate entries; instead, data collectors instructed participants to complete the questionnaire only once. No submissions with atypical timestamps were identified. Because the survey link was distributed openly, the number of individuals who received or viewed the invitation is unknown, and viewing and participation rates could not be calculated.

### Questionnaire Validity

The questionnaire underwent a multistep validation process to ensure clarity, relevance, and alignment with the study constructs. Face validity was assessed through a pilot test with 14 medical students and early graduates, who evaluated the questionnaire for comprehensibility, wording clarity, and response burden. Minor adjustments were made to item phrasing and formatting based on participant feedback to optimize ease of completion and alignment with respondents’ understanding. The 14 pilot participants were excluded from the final analytic sample (N=432) to avoid potential bias.

### Ethical Considerations

Electronic informed consent was collected from all study participants before they could access the questionnaire. Ethical approval was obtained from the research ethics committee of Nafeer Elafia Hospital, the Northern State Ministry of Health, Northern State, Sudan (reference number 300101; approved on January 30, 2025). The study was conducted according to the principles of the Declaration of Helsinki, and all responses were collected anonymously.

### Statistical Analysis

Data were analyzed using R software (version 4.4.2; R Foundation for Statistical Computing). Descriptive statistics were used to summarize demographic characteristics, social media usage patterns, and responses to questionnaire items, with results reported as frequencies and percentages.

For the attitudinal section assessing perceptions of professionalism and professional identity on social media, responses were numerically coded according to the predefined scoring scheme, and a total professionalism attitude score was generated. Items in the *Experiences and Behaviors* domain and the *Benefits and Risks Awareness* domain were treated as independent categorical indicators and summarized descriptively, as they did not represent a single latent construct suitable for scale formation.

The construct validity of the professionalism attitude scale was assessed using EFA. Data suitability was confirmed with the Kaiser-Meyer-Olkin measure of sampling adequacy and the Bartlett test of sphericity; the number of factors to retain was determined by parallel analysis, and factors were extracted using the minimum residual (MINRES) method with oblimin rotation. Full details are provided in Multimedia Appendix 1.

Bivariate analyses using Pearson chi-square tests were conducted to explore associations between 3 primary demographic characteristics (sex, age group, and current status) and key social media behaviors and perceptions: heavy usage (≥5 h/d), following health care pages, posting health care content, confidence in posting appropriateness, and experiencing negative outcomes. Chi-square statistics are reported with their degrees of freedom. Because these bivariate tests were exploratory, unadjusted *P* values are reported and no formal correction for multiple testing was applied; instead, multivariable logistic regression was used to control for confounding and to reduce reliance on unadjusted bivariate comparisons.

Multivariable logistic regression models were then fitted for each of the same 5 binary outcomes to identify independent demographic and behavioral predictors. All models included sex, age (dichotomized as ≤25 y vs >25 y), current status (with medical student as the reference), and maintenance of separate personal and professional accounts as predictors; relevant behavioral covariates (daily usage, following health care pages, confidence in post appropriateness, and posting health care content) were additionally included when conceptually appropriate for the outcome being modeled. Results are reported as odds ratios (ORs) with 95% CIs. A significance level of *P* less than .05 was used for all statistical tests.

## Results

### Demographic Characteristics

A total of 432 participants were included in the study. Most were aged 26 to 30 years (n=197, 45.6%), followed by 21 to 25 years (n=181, 41.9%), with smaller proportions in the younger and older age groups. Females represented 52.3% (n=226) of the sample. Participants’ professional status included medical students (n=135, 31.3%), interns (n=110, 25.5%), early graduates in practice (n=95, 21.9%), and residents (n=92, 21.3%). Among the 135 medical students, the largest representation came from Bahri University (n=45, 33.3%), followed by Elrazi University (n=17, 12.6%), Khartoum University (n=13, 9.6%), and National University (n=11, 8.1%). Students were distributed across academic years, with the highest proportions in the fifth (n=40, 29.6%) and sixth (n=41, 30.4%) years (Table 1).

**Table 1.** Demographic characteristics of study participants (N=432).

Characteristic	Values, n (%)
Sex	
Female	226 (52.3)
Male	206 (47.7)
Age group (y)	
16‐20	14 (3.2)
21‐25	181 (41.9)
26‐30	197 (45.6)
31‐35	33 (7.6)
36‐40	7 (1.6)
Current status	
Medical student	135 (31.3)
Intern	110 (25.5)
Early graduate in practice	95 (21.9)
Resident	92 (21.3)
University (medical students, n=135)	
Ahfad University	5 (3.7)
Al-Neelain University	2 (1.5)
Alzaim Alazhary University	1 (0.7)
Bahri University	45 (33.3)
Dongola University	2 (1.5)
El-Fagr University	2 (1.5)
Elrazi University	17 (12.6)
Gezira University	1 (0.7)
Ibn Sina University	1 (0.7)
Kassala University	1 (0.7)
Khartoum University	13 (9.6)
Kordofan University	4 (2.9)
National Ribat University	8 (5.9)
National University	11 (8.1)
Sudan International University	6 (4.4)
Sudan University of Science and Technology	6 (4.4)
University of El Imam El Mahdi	2 (1.5)
University of Medical Science and Technology	8 (5.9)
Academic year (medical students, n=135)	
First	5 (3.7)
Second	4 (2.9)
Third	16 (11.9)
Fourth	29 (21.5)
Fifth	40 (29.6)
Sixth	41 (30.4)

### Social Media Usage Patterns

Of the 432 participants, WhatsApp, Instagram (Meta Platforms, Inc.), and Facebook (Meta Platforms, Inc.) were the most commonly used platforms, reported by 322 (74.5%), 276 (63.9%), and 211 (48.8%) participants, respectively. TikTok (ByteDance Ltd) and X (formerly Twitter) were less frequently used (Figure S1 in Multimedia Appendix 3). Daily usage was high, with 179 (41.4%) participants spending at least 5 hours per day on social media and another 164 (37.9%) spending 3 to 4 hours per day. Most participants followed health care–related pages (n=381, 88.2%) and reported varying frequencies of posting health care–related content: rarely (n=139, 32.2%), sometimes (n=127, 29.4%), never (n=119, 27.5%), and frequently (n=47, 10.9%; Table 2). Participants primarily used social media for educational purposes (n=335, 77.5%), as well as for entertainment (n=287, 66.4%) and networking (n=241, 55.8%). About one-quarter (n=115, 26.6%) used social media for patient communication or public health advocacy (Figure S2 in Multimedia Appendix 3).

**Table 2.** Social media usage patterns among participants (N=432).

Items and responses	Values, n (%)
Average daily time spent on social media (h)	
Less than 1	14 (3.2)
1‐2	75 (17.4)
3‐4	164 (37.9)
5 or more	179 (41.4)
Follows health care–related pages, professionals, or organizations	
Yes	381 (88.2)
No	51 (11.8)
Posts health care–related content (eg, medical news, infographics, or opinions)	
Frequently	47 (10.9)
Sometimes	127 (29.4)
Rarely	139 (32.2)
Never	119 (27.5)

### Reliability and Construct Validity of the Professionalism Attitude Scale

The EFA supported a unidimensional structure: the single extracted factor explained 26.0% of the total variance with excellent model fit (*χ*²_₅_=2.2, *P*=.82; Tucker-Lewis Index=1.02; root mean square error of approximation=0.0, 90% CI 0.00‐0.04). Four of the 5 items showed salient loadings (≥0.40) on this factor, whereas the behavioral item on modifying or removing a post did not load on the attitudinal construct and was excluded from the final validated scale (Multimedia Appendix 1). Following the EFA, internal consistency was assessed. The initial 5-item scale demonstrated a raw Cronbach α of 0.60, and item-total statistics supported the EFA findings, indicating that removal of the behavioral item would improve the standardized internal consistency. The final validated 4-item scale demonstrated moderate reliability (raw Cronbach α=0.60 and standardized Cronbach α=0.62), which is considered acceptable for a brief scale capturing the underlying professionalism attitude construct (Multimedia Appendix 2).

### Attitudes Toward Professionalism and Professional Identity

Of the 432 participants, attitudes toward digital professionalism were generally positive. More than half agreed that their social media presence reflects their identity as current or future health care professionals, including 87 (20.1%) who strongly agreed and 138 (31.9%) who agreed, while only 74 (17.1%) disagreed or strongly disagreed. Most participants recognized the importance of maintaining a professional online image, with 199 (46.1%) rating it as very important and 191 (44.2%) as somewhat important. Nearly half of the participants (n=199, 46.1%) had modified or removed a post to align with professional expectations. Perceptions of social media’s broader impact were also favorable: 302 (69.9%) participants agreed or strongly agreed that social media influences public trust in health care professionals, and an equal proportion (n=302, 69.9%) believed it enhances their professional reputation (Table 3).

**Table 3.** Attitudes toward professional identity and professionalism on social media (N=432).

Items and responses	Values, n (%)
Social media presence reflects professional identity	
Strongly agree	87 (20.1)
Agree	138 (31.9)
Neutral	133 (30.8)
Disagree	50 (11.6)
Strongly disagree	24 (5.6)
Importance of professional image online	
Very important	199 (46.1)
Somewhat important	191 (44.2)
Not important	42 (9.7)
Social media influences public trust	
Strongly agree	110 (25.5)
Agree	192 (44.4)
Neutral	108 (25)
Disagree	16 (3.7)
Strongly disagree	6 (1.4)
Engaging on social media enhances professional reputation	
Yes	302 (69.9)
No	130 (30.1)
Modified or removed a post for professional reasons	
Yes	199 (46.1)
No	233 (53.9)

### Experiences and Online Behaviors

Among the 432 participants, experiences related to professionalism on social media varied. Over one-third (n=153, 35.4%) had experienced a negative outcome related to their social media activity. Confidence in distinguishing appropriate from inappropriate posts was generally high, with 261 (60.4%) participants expressing confidence, though 131 (30.3%) reported only sometimes feeling confident and 40 (9.3%) reported no confidence. Exposure to unprofessional content was relatively common: 142 (32.9%) participants had encountered a health care professional posting inappropriate material online. Regarding account management practices, 128 (29.6%) participants maintained separate personal and professional accounts, 175 (40.5%) did not, and 129 (29.9%) were planning to do so (Table 4).

**Table 4.** Experiences and online behaviors related to social media use (N=432).

Items and responses	Values, n (%)
Experienced a negative outcome due to social media	
Yes	153 (35.4)
No	279 (64.6)
Confidence in distinguishing appropriate posts	
Yes	261 (60.4)
Sometimes	131 (30.3)
No	40 (9.3)
Encountered a health care professional posting inappropriate content	
Yes	142 (32.9)
No	290 (67.1)
Maintains separate personal and professional accounts	
Yes	128 (29.6)
No	175 (40.5)
Planning to	129 (29.9)

### Perceived Benefits and Risks of Social Media Use in a Health Care Context

Among the 432 participants, several professional advantages of using social media were identified. The most frequently reported benefit was access to updated medical knowledge (n=321, 74.3%), followed by opportunities for career development (n=260, 60.2%), and professional networking (n=235, 54.4%). A substantial proportion also highlighted the role of social media in public health advocacy (n=177, 40.9%) and enhancing visibility or recognition as a health care professional (n=150, 34.7%). These findings indicate that participants largely view social media as a platform that supports both learning and career progression (Figure S3 in Multimedia Appendix 3).

Among the 432 participants, a range of concerns regarding the use of social media in health care was reported. The most frequently cited risk was the spread of misinformation (n=287, 66.4%), followed by fears of negative public perception (n=199, 46.1%), and potential breaches of patient confidentiality (n=198, 45.8%). Additional concerns included blurred personal-professional boundaries (n=142, 32.9%), job-related consequences (n=111, 25.7%), and experiences with or fears of cyberbullying or trolling (n=101, 23.4%). These results illustrate a balanced understanding among participants of both the professional opportunities and the potential hazards associated with social media use (Figure S4 in Multimedia Appendix 3).

### Demographic Associations With Social Media Behaviors and Outcomes

Bivariate analyses using Pearson chi-square tests examined associations between respondent characteristics (sex, age, and current professional status) and social media behaviors, confidence, and experiences (Table 5).

**Table 5.** Associations between demographic characteristics and key social media behaviors (N=432).

Characteristic	≥5 hours/day	Follows health care pages	Posts health care content	Confident in appropriateness	Negative outcome
Sex					
Female (n=226), n (%)	109 (48.2)	201 (88.9)	170 (75.2)	127 (56.2)	68 (30.1)
Male (n=206), n (%)	70 (33.9)	180 (87.4)	143 (69.4)	134 (65.0)	85 (41.3)
Chi-square (*df*); *P* value**^a^**	8.44 (1); .004	0.12 (1); .73	1.54 (1); .22	3.17 (1); .08	5.40 (1); .02
Age group (y)					
16‐20 (n=14), n (%)	4 (28.6)	10 (71.4)	8 (57.1)	9 (64.3)	4 (28.6)
21‐25 (n=181), n (%)	72 (39.8)	160 (88.4)	118 (65.2)	110 (60.8)	67 (37.0)
26‐30 (n=197), n (%)	92 (46.7)	176 (89.3)	157 (76.7)	114 (57.9)	67 (34.0)
31‐35 (n=33), n (%)	7 (21.2)	28 (84.8)	26 (78.8)	23 (69.7)	14 (42.4)
36‐40 (n=7), n (%)	4 (57.1)	7 (100)	4 (57.1)	5 (71.4)	1 (14.3)
Chi-square (*df*); *P* value**^a^**	9.68 (4); .046	5.33 (4); .26	13.09 (4); .01	2.18 (4); .70	2.73 (4); .60
Current status					
Medical student (n=135), n (%)	55 (40.7)	120 (88.9)	89 (65.9)	73 (54.1)	49 (36.3)
Intern (n=110), n (%)	49 (44.5)	98 (89.1)	83 (75.5)	74 (67.3)	35 (31.8)
Early graduate (n=95), n (%)	40 (42.1)	84 (88.4)	71 (74.7)	58 (61.1)	34 (35.8)
Resident (n=92), n (%)	35 (38.0)	79 (85.9)	70 (76.1)	56 (60.9)	35 (38.0)
Chi-square (*df*); *P* value**^a^**	0.92 (3); .82	0.63 (3); .89	4.24 (3); .24	4.46 (3); .22	0.95 (3); .81

a Pearson chi-square test; values shown are the chi-square statistic (df) followed by the *P* value.

Daily social media use varied significantly across demographic factors. A significant association was observed between sex and the duration of daily social media use (*χ*²_₁_=8.44; *P*=.004): a higher proportion of female respondents (109/226, 48.2%) reported using social media for 5 or more hours per day compared with male respondents (70/206, 33.9%). Daily usage was also significantly associated with age (*χ*²_₄_=9.68; *P*=.046), with the highest proportion of heavy users (≥5 h/d) concentrated among respondents aged 26 to 30 years (92/197, 46.7%). Current professional status was not significantly associated with daily usage duration (*P*=.82).

The vast majority of the sample engaged with professional content, with 381 of 432 participants (88.2%) reporting that they follow health care pages. This behavior was consistent across the cohort, showing no significant differences based on sex, age, or current status (*P*>.05 for all). In contrast, active content creation was influenced by age. While there were no significant differences in posting behavior by sex (*P*=.22) or current status (*P*=.24), posting health care–related content was significantly associated with age (*χ*²_₄_=13.09; *P*=.01): respondents aged 26 to 30 years were the most active creators, with 157 of 197 (79.7%) reporting that they post health care content, compared with 118 of 181 (65.2%) of those aged 21 to 25 years.

Respondents’ confidence in the appropriateness of their posts did not show any statistically significant associations across demographic groups, although male respondents reported slightly higher confidence (134/206, 65.0%) compared with female respondents (127/226, 56.2%; *χ*²_₁_=3.17; *P*=.08). However, when examining adverse digital experiences, a significant association with sex was identified (*χ*²_₁_=5.40; *P*=.02): male respondents were more likely to report having experienced a negative outcome on social media (85/206, 41.3%) compared with their female counterparts (68/226, 30.1%). Neither age (*P*=.60) nor current professional status (*P*=.81) was significantly associated with experiencing negative digital outcomes.

### Multivariable Predictors of Social Media Behaviors and Outcomes

Multivariable logistic regression models examined the demographic and behavioral predictors of 5 distinct social media outcomes: heavy usage (≥5 h/d), following health care pages, posting health care content, confidence in the appropriateness of posting, and experiencing negative outcomes (Table 6).

**Table 6.** Multivariable logistic regression analysis of factors associated with social media behaviors and outcomes (N=432)^a^.

Predictor	≥5 hours/day, OR^b^ (95% CI); *P* value	Follows pages, OR (95% CI); *P* value	Posts content, OR (95% CI); *P* value	Confident, OR (95% CI); *P* value	Negative outcome, OR (95% CI); *P* value
Sex (reference: female)					
Male	0.54 (0.36‐0.81); .003	0.86 (0.47‐1.58); .63	0.66 (0.41‐1.04); .08	1.38 (0.93‐2.07); .11	2.12 (1.36‐3.33); <.001
Age (reference: ≤25 y**)**					
>25 years	1.31 (0.82‐2.08); .26	1.46 (0.73‐2.91); .28	2.03 (1.19‐3.49); .01	0.73 (0.46‐1.17); .20	0.76 (0.46‐1.25); .28
Current status (reference: medical student)					
Early graduate	1.04 (0.58‐1.88); .89	0.84 (0.34‐2.10); .70	1.21 (0.62‐2.39); .58	1.46 (0.81‐2.64); .21	0.92 (0.48‐1.72); .78
Intern	1.18 (0.67‐2.08); .58	0.91 (0.38‐2.21); .83	1.24 (0.64‐2.40); .53	1.98 (1.11‐3.59); .02	0.82 (0.44‐1.53); .54
Resident	0.88 (0.46‐1.68); .71	0.63 (0.24‐1.64); .34	1.25 (0.59‐2.71); .56	1.47 (0.77‐2.80); .24	1.04 (0.52‐2.07); .91
Separate accounts (reference: no)					
Planning to	1.12 (0.70‐1.80); .63	1.26 (0.61‐2.69); .54	1.25 (0.73‐2.18); .42	1.03 (0.65‐1.65); .90	2.03 (1.20‐3.47); .008
Yes	1.02 (0.63‐1.64); .94	1.00 (0.50‐2.03); *P*≥.99	1.52 (0.87‐2.69); .15	1.75 (1.08‐2.86); .02	4.13 (2.47‐7.00); <.001
Uses social media ≥5 hours/day (reference: no)					
Yes	—^c^	0.73 (0.40‐1.33); .30	—	—	1.29 (0.83‐2.00); .25
Follows health care pages (reference: no)					
Yes	—	—	5.71 (3.03‐11.10); <.001	—	—
Confident in posts (reference: no)					
Yes	—	—	1.76 (1.10‐2.80); .02	—	0.74 (0.47‐1.15); .18
Posts health care content (reference: no)					
Yes	—	—	—	—	2.64 (1.58‐4.52); <.001

a Each column represents a separate multivariable model adjusted for all predictors, with values shown in that column.

b OR: odds ratio.

c Variable was not included as a predictor in that specific regression model.

When examining daily social media time, male participants were significantly less likely than female participants to report use of 5 hours per day or more (OR 0.54, 95% CI 0.36‐0.81; *P*=.003). Age, current training status, and the separation of personal and professional accounts were not significant predictors of heavy usage. The model predicting whether participants followed health care–related pages yielded no statistically significant demographic or behavioral predictors.

Several factors significantly predicted the likelihood of posting health care–related content. Participants older than 25 years had twice the odds of posting content compared with those aged 25 years and younger (OR 2.03, 95% CI 1.19‐3.49; *P*=.01). Behavioral factors were also strong predictors: those who followed health care pages were nearly 6 times more likely to post their own content (OR 5.71, 95% CI 3.03‐11.10; *P*<.001), and individuals who reported confidence in the appropriateness of their posts had significantly higher odds of actively posting content (OR 1.76, 95% CI 1.10‐2.80; *P*=.02).

Current training status and account management strategies were significantly associated with confidence in the appropriateness of social media posts. Compared with medical students, interns were nearly twice as likely to report feeling confident (OR 1.98, 95% CI 1.11‐3.59; *P*=.02). Participants who actively maintained separate personal and professional accounts also demonstrated significantly higher odds of confidence than those who did not separate their accounts (OR 1.75, 95% CI 1.08‐2.86; *P*=.02).

The model identified multiple significant predictors of experiencing negative outcomes related to social media use. Male participants had more than twice the odds of reporting a negative outcome compared with their female counterparts (OR 2.12, 95% CI 1.36‐3.33; *P*<.001). Account separation also played a substantial role: paradoxically, participants who already maintained separate accounts had more than 4 times the odds of experiencing a negative outcome (OR 4.13, 95% CI 2.47‐7.00; *P*<.001), and those planning to separate their accounts had twice the odds (OR 2.03, 95% CI 1.20‐3.47; *P*=.008) compared with those who did not separate them. Finally, posting health care content was a strong independent predictor, significantly increasing the likelihood of experiencing a negative outcome (OR 2.64, 95% CI 1.58‐4.52; *P*<.001).

## Discussion

### Principal Findings

In this cross-sectional survey of 432 medical students and early-career doctors in Sudan, we found high levels of social media use, with 179 (41.4%) participants online for at least 5 hours daily, alongside generally positive attitudes toward digital professionalism, growing awareness of the professional implications of online activity, and substantial gaps in training and institutional guidance. Female participants reported heavier daily use, whereas male participants and those posting health care–related content had significantly higher odds of experiencing negative online outcomes. To our knowledge, this is the first study to examine digital professionalism and professional identity among medical students and early-career doctors in Sudan. It advances the existing literature, which has been largely descriptive and grounded in Western educational contexts [7,8,10], in 2 ways: by combining a psychometrically evaluated attitude scale with multivariable modeling of the predictors of key online behaviors and outcomes and by providing empirical evidence from a collectivist, resource-constrained setting where cultural norms around reputation and hierarchy shape how professional identity is negotiated online.

### Comparison With Prior Work

Our finding of social media platforms usage for at least 5 hours per day aligns with the findings of Faihs et al [15], who reported an increasing interest among medical students in learning more about digital technologies, reflecting their immersion in digital ecosystems. Moreover, Gagnon and Sabus [16] reflected on the increase in the use of social media by health care providers and the need to propose guidelines for social media usage by physical therapy professionals, which indicates the need to maintain patients’ privacy and adhere to the principles of medical ethics even when social media is used for educational purposes. The study participants, including both undergraduates and postgraduates, reported growing trends toward the use of social media in informal learning activities besides knowledge sharing and networking. In contrast, Sterling et al [4] reported that there is inconclusive evidence on the impact of social media on graduate medical education and professionalism despite its common use. Around half of the study participants (225/432, 52.1%) agreed that their social media accounts reflect their professional identity, emphasizing that digital spaces have become essential in shaping identity and supporting the findings of Wu et al [17] that social media shapes professional identity among medical students through informational and emotional support. Out of 432 participants, 199 (46.1%) had incidents where they removed or edited their social media posts because of considerations of their professional role, while 233 (53.9%) had not. These differences indicate differences in the perception and management of the professional implications of their online engagement. While the reasons for these behaviors were not directly measured in this study, one possible explanation is that those who removed or edited their content had greater awareness of the professional and reputational consequences of their posts, while those who did not may have perceived their content as appropriate or were less aware of potential professional consequences. While this interpretation remains speculative, the variation in practices is consistent with an absence of systematic training or institutional policies on professional conduct on social media. Ezeilo et al [18] reported that social media has been widely used in health advocacy and awareness-raising, while only 115 (26.6%) participants used social media for advocacy and public health promotion. This suggests that medical students and early-career doctors are not fully engaging with social media for advocacy, perhaps due to perceived gaps in social accountability, training, or confidence. Interestingly, 302 (69.9%) participants believed that social media engagement can improve professional reputation, consistent with the study by Ferrell and Campos-Castillo [19], which suggested that tackling misinformation on X and engaging with the public can improve credibility. Participants also expressed concerns regarding social media practices that negatively impact them as medical students and early-career doctors, such as the spread of misinformation (n=287, 66.4%), negative public perception (n=199, 46.1%), and breaches of patient confidentiality (n=198, 45.8%). These concerns reflect longstanding issues outlined in the literature warning that digital platforms can amplify unprofessional conduct, expose trainees to reputational harm, and diminish public trust in the medical profession [20]. Although many participants reported confidence in distinguishing professional from unprofessional content, the confidence level was not associated with the level of education or training. Because curricular exposure to digital professionalism was not directly assessed in this study, this interpretation is hypothetical; nevertheless, the absence of a gradient across training stages would be consistent with limited formal education on the influence of social media on medical professionalism. Approximately one-third of respondents (n=142, 32.9%) reported encountering inappropriate content shared by health care professionals, a finding consistent with Kitsis et al [12], who reported that medical students engaged in sharing unprofessional content on social media.

### Implications for Medical Education and Practice

The findings carry important implications for medical education and professional regulation. There is a need for structured curricula that address topics such as digital professionalism, ethical online behavior, management of digital identity, and responsible engagement in public communication. In addition, given the frequent use of social media for medical learning by participants, educators and institutions could leverage social media platforms to disseminate evidence-based content and create supportive online learning communities. Clear institutional guidelines, mentorship, and support systems to address cyberbullying and negative online experiences would also help in promoting safe and professional use of digital environments.

### Limitations

There are several limitations to this study that should be considered. The cross-sectional design limits the ability to follow changes in attitudes and behaviors over time. Self-reported data may be subject to recall bias and the desire to provide socially acceptable answers, particularly around professionalism. The sample, while large, was a convenience sample obtained through an online questionnaire; this form of sampling is prone to self-selection bias and is likely to overrepresent those who are already active social media users, which may inflate estimates of usage intensity and engagement and limit the generalizability of the results to all medical students and early-career health professionals in Sudan. In addition, the inclusion of professionals who graduated up to 10 years previously introduces some heterogeneity in experience, although current status (student, intern, early graduate, or resident) was accounted for in the stratified and multivariable analyses. Despite these limitations, the study offers valuable insights into the perceptions of emerging health care professionals regarding the junction of social media and professional identity. Future research should investigate longitudinal changes in digital behavior and professionalism throughout medical training.

### Conclusions

This study highlights the growing significance of social media in the daily lives and professional journeys of medical students and early-career doctors in Sudan. Participants acknowledged the educational and networking opportunities offered by these platforms, while also being aware of the ethical risks and possible impacts on their professional identities. Although most respondents demonstrated awareness of professional norms by editing or deleting inappropriate posts and recognizing the effect of online behavior on public confidence, practices varied, indicating significant gaps in training and institutional support. The noted sex differences, with female participants reporting higher daily usage and male participants experiencing more frequent negative online experiences, highlight the complex navigation of professional identity within digital spaces. The results demonstrate the value of systematic and context-sensitive education in digital professionalism within medical curricula. This kind of education needs to be more comprehensive than rule-based education and should encompass reflective judgment, responsible online behavior, and confidence in the management of one’s digital identity. Therefore, institutions can prepare future health care professionals to participate in digital environments safely, ethically, and meaningfully.

## Supplementary material

10.2196/89260 Multimedia Appendix 1 Exploratory factor analysis and construct validity of the professionalism attitude scale.

10.2196/89260 Multimedia Appendix 2 Internal consistency reliability analysis of the professionalism attitude scale.

10.2196/89260 Multimedia Appendix 3 Supplementary figures showing participants’ perceived risks and benefits of social media use in health care, purposes of social media use, and regularly used social media platforms (N=432).

10.2196/89260 Checklist 1 CHERRIES checklist.

## Abbreviations

CHERRIES: Checklist for Reporting Results of Internet E-Surveys
EFA: exploratory factor analysis
MINRES: minimum residual
OR: odds ratio
PIF: professional identity formation
