# Supplementary material for: Social Media Use, Professionalism, and Professional Identity Among Medical Students and Early-Career Health Care Graduates in Sudan: Cross-Sectional Survey Study
Source: Online J Public Health Inform. 2026 Aug 25;18:e89260. doi: 10.2196/89260 (PMC13505479; doi:10.2196/89260)
Supplement: Supplementary file 1 [file ojphi-v18-e89260-s001.docx]

**Multimedia Appendix 1**

**Exploratory Factor Analysis and Construct Validity of the Professionalism Attitude Scale**

An exploratory factor analysis (EFA) was conducted to evaluate the construct validity of the Professionalism Attitude Scale. Prior to extraction, data suitability was confirmed via the Kaiser-Meyer-Olkin measure of sampling adequacy and the Bartlett test of sphericity.

To determine the optimal number of factors to retain, a parallel analysis was executed. Visual inspection of the resulting scree plot revealed that only the first principal factor yielded an eigenvalue substantially greater than those generated from randomly simulated and resampled data matrices. Consequently, a unidimensional structure was supported and extracted using the minimum residual (minres) method with an oblimin rotation.

The resulting single-factor model explained 26.0% of the total variance and demonstrated an excellent fit to the data (χ²₅=2.2, P=.82; Tucker-Lewis index [TLI]=1.02; root mean square error of approximation [RMSEA]=0.00, 90% CI 0.00-0.04). Four of the five scale items demonstrated strong salient loadings (≥0.40) onto this single factor, representing a cohesive “Professionalism Attitude” construct. The item regarding behavioral modification of a post (“Have you ever modified or removed a post...”) failed to meet the 0.30 salient loading threshold, statistically differentiating this behavioral action from the underlying attitudinal construct. As a result, the final validated attitude scale consists of four highly correlated items.

**Table A1.** Factor loadings, communalities, and model fit indices for the Professionalism Attitude Scale.

| **Item** | **Factor loading** | **Communality (h²)** |
| --- | --- | --- |
| Social media reflects professional identity | 0.72 | 0.51 |
| Importance of online professional image | 0.58 | 0.34 |
| Social media influences public trust | 0.47 | 0.22 |
| Social media enhances reputation | 0.40 | 0.16 |
| Modified or removed a post for professionalism | —^a^ | 0.07 |
| **Model fit indices** |  |  |
| Tucker-Lewis index | 1.02 |  |
| RMSEA^b^ (90% CI) | 0.00 (0.00-0.04) |  |
| Model chi-square (P value) | 2.2 (P=.82) |  |
| Variance explained | 26.0% |  |

^a^Loading below the 0.30 salient threshold (item excluded from the final scale).

^b^RMSEA: root mean square error of approximation.
