# Supplementary material for: Social Media Use, Professionalism, and Professional Identity Among Medical Students and Early-Career Health Care Graduates in Sudan: Cross-Sectional Survey Study
Source: Online J Public Health Inform. 2026 Aug 25;18:e89260. doi: 10.2196/89260 (PMC13505479; doi:10.2196/89260)
Supplement: Supplementary file 2 [file ojphi-v18-e89260-s002.docx]

**Multimedia Appendix 2**

**Internal Consistency Reliability Analysis of the Professionalism Attitude Scale**

Following the exploratory factor analysis, Cronbach α was calculated to assess the internal consistency of the Professionalism Attitude Scale. The initial 5-item scale demonstrated a raw Cronbach α of .60. Item-total statistics supported the exploratory factor analysis findings, indicating that the removal of the behavioral item concerning modifying or removing a post would improve the scale’s standardized internal consistency. The final validated 4-item attitudinal scale demonstrated moderate reliability, with a raw Cronbach α of .60 and a standardized α of .62. Given the brief nature of the 4-item scale, this level of internal consistency is considered acceptable for capturing the underlying professionalism attitude construct.

**Table A2.** Internal consistency reliability analysis of the Professionalism Attitude Scale.

| **Scale item** | **Cronbach α if item deleted** |
| --- | --- |
| **Initial 5-item scale (overall α=.60)** |  |
| Social media reflects professional identity | .46 |
| Importance of online professional image | .50 |
| Social media influences public trust | .53 |
| Social media enhances reputation | .57 |
| Modified or removed a post for professionalism | .60 |
| **Final validated 4-item scale (overall α=.60; standardized α=.62)** |  |
