# Supplementary material for: Social Media Use, Professionalism, and Professional Identity Among Medical Students and Early-Career Health Care Graduates in Sudan: Cross-Sectional Survey Study
Source: Online J Public Health Inform. 2026 Aug 25;18:e89260. doi: 10.2196/89260 (PMC13505479; doi:10.2196/89260)
Supplement: Supplementary file 3 [file ojphi-v18-e89260-s003.docx]

Figure S1. Social media platforms regularly used by health care students and professionals (N=432). Values are the percentage of participants reporting regular use of each platform; multiple responses were allowed.

Figure S2. Purposes of social media use among participants (N=432). Values are the percentage of participants selecting each purpose; multiple responses were allowed.

Figure S3. Perceived benefits of social media use as a health care student or professional (N=432). Values are the percentage of participants selecting each benefit; multiple responses were allowed. Figure S4. Perceived risks of social media use in a health care context (N=432). Values are the percentage of participants selecting each risk; multiple responses were allowed.
