# Supplementary material for: Social Media Use, Professionalism, and Professional Identity Among Medical Students and Early-Career Health Care Graduates in Sudan: Cross-Sectional Survey Study
Source: Online J Public Health Inform. 2026 Aug 25;18:e89260. doi: 10.2196/89260 (PMC13505479; doi:10.2196/89260)
Supplement: Supplementary file 4 [file ojphi-v18-e89260-s004.docx]

**Checklist 1:**

**CHERRIES (Checklist for Reporting Results of Internet E-Surveys) Checklist**

All section references refer to the revised manuscript.

| **Item category** | **Checklist item** | **Explanation / where reported** |
| --- | --- | --- |
| **Design** | Describe survey design | Cross-sectional online survey of a convenience sample of medical students and early-career health professionals in Sudan (Methods, Study Design and Setting; Study Population and Sampling). |
| **IRB approval and informed consent** | IRB approval | Approved by the research ethics committee of Nafeer Elafia Hospital, Northern State Ministry of Health, Sudan (reference number 300101; approved on January 30, 2025) (Methods, Ethical Considerations). |
|  | Informed consent | Electronic informed consent obtained from all participants on the first page of the questionnaire before accessing the survey items (Methods, Ethical Considerations; Data Collection Tool and Procedure). |
|  | Data protection | Responses were collected anonymously; no identifying information was gathered (Methods, Ethical Considerations). |
| **Development and pretesting** | Development and testing | Questionnaire developed specifically for this study by the research team, informed by existing literature; face validity assessed through a pilot test with 14 medical students and early graduates (excluded from the final sample), with adjustments to wording and formatting (Methods, Data Collection Tool and Procedure; Questionnaire Validity). |
| **Recruitment process** | Open vs closed survey | Open survey: the survey link was open to anyone who received it (Methods, Study Population and Sampling). |
|  | Contact mode | Participants were contacted and invited online via medical social media groups and WhatsApp groups dedicated mainly to medical students and healthcare professionals (Methods, Study Population and Sampling). |
|  | Advertising the survey | The survey was distributed in medical social media groups and WhatsApp groups dedicated mainly to medical students and healthcare professionals (Methods, Study Population and Sampling). |
| **Survey administration** | Web/email survey | Anonymous, structured, self-administered online questionnaire in English, administered via Google Forms (Methods, Data Collection Tool and Procedure). |
|  | Context | Conducted June-August 2025 among medical students and early-career health professionals in Sudan (Methods, Study Design and Setting). |
|  | Mandatory/voluntary | Participation was voluntary (Methods, Study Population and Sampling). |
|  | Incentives | No incentives were offered (Methods, Study Population and Sampling). |
|  | Time/date | Data collected from June to August 2025 (Methods, Study Design and Setting). |
|  | Randomization of items | No randomization was used; items and response options were presented in a fixed order for all respondents (Methods, Data Collection Tool and Procedure). |
|  | Adaptive questioning | No adaptive questioning was used (Methods, Data Collection Tool and Procedure). |
|  | Number of items | Six sections: demographics (6 items), 5 attitudinal items, 7 experience/behavior items, and 2 checklist items, plus student-specific items (eg, university, academic year) and doctor-specific items (eg, public or private hospital) (Methods, Data Collection Tool and Procedure). |
|  | Number of screens (pages) | Five pages: (1) informed consent; (2) demographic characteristics and career level (undergraduate/postgraduate); (3) medical student items; (4) practicing doctor items; (5) attitude, behavior, and checklist items (Methods, Data Collection Tool and Procedure). |
|  | Completeness check | All items were mandatory, so incomplete questionnaires could not be submitted (Methods, Data Collection Tool and Procedure). |
|  | Review step | Respondents were unable to change their answers after submission (Methods, Data Collection Tool and Procedure). |
| **Response rates** | Unique site visitor | Not assessed; Google Forms does not record unique visitors, and the survey used an openly distributed link (Methods, Data Collection Tool and Procedure). |
|  | View rate / participation rate / completion rate | View and participation rates could not be calculated because the survey link was distributed openly and the number of individuals who received or viewed the invitation is unknown. Because all items were mandatory, all 432 submitted questionnaires were complete (Methods, Data Collection Tool and Procedure). |
| **Preventing multiple entries** | Cookies / IP check / log file analysis | No technical measures (cookies, IP address checks, or log file analysis) were used to prevent duplicate entries; data collectors instructed participants to complete the questionnaire only once (Methods, Data Collection Tool and Procedure). |
| **Analysis** | Handling of incomplete questionnaires | Only complete questionnaires were recorded and analyzed, as all items were mandatory before submission; all 432 submitted responses were analyzed (Methods, Data Collection Tool and Procedure; Statistical Analysis). |
|  | Questionnaires submitted with an atypical timestamp | No submissions with atypical timestamps were identified, and no timestamp-based exclusions were applied (Methods, Data Collection Tool and Procedure). |
|  | Statistical correction | No weighting or propensity score adjustment was applied; results are unadjusted for sampling (Methods, Statistical Analysis). |
